# Pain in the three spinal regions: the same disorder? Data from a population-based sample of 34,902 Danish adults

**DOI:** 10.1186/2045-709X-20-11

**Published:** 2012-04-05

**Authors:** Charlotte Leboeuf-Yde, René Fejer, Jan Nielsen, Kirsten O Kyvik, Jan Hartvigsen

**Affiliations:** 1 The Research Department, the Spine Centre of Southern Denmark, Hospital Lillebaelt, Middelfart, Denmark; 2 Institute of Regional Health Services Research, Faculty of Health Sciences, University of Southern Denmark, Odense, Denmark; 3 Odense Patient data Exploratory Network (OPEN), Odense University Hospital, Odense, Denmark; 4 Institute of Sport Science and Clinical Biomechanics, Faculty of Health Sciences, University of Southern Denmark, Odense, Denmark; 5 Nordic Institute of Chiropractic and Clinical Biomechanics, Odense, Denmark; 6 The Spine Centre of Southern Denmark, Hospital Lillebaelt, Middelfart, Ostre Hougvej 55, DK-5500 Middelfart, Denmark

## Abstract

**Background:**

Studies of back pain are typically based on the assumption that symptoms from different parts of the spine are distinctive entities. Recently, however, the assumption that back pain is a site-specific disorder has been challenged, suggesting that localized back pain should be seen as part of a general musculoskeletal syndrome.

**Objectives:**

To describe and compare the patterns of reporting of pain and consequences of pain in the three spinal regions.

**Methods:**

In all, 34,902 (74%) twin individuals representative of the general Danish population, aged 20 to 71, participated in a cross-sectional nation-wide survey. Identical questions from the Standardised Nordic Questionnaire for each of the three spinal regions were used for lumbar, mid-back and neck pain respectively: Pain past year, pain ever, radiating pain, and consequences of back pain (care-seeking, reduced physical activities, sick-leave, change of work/work duties and disability pension). The relative prevalence estimates of these variables were compared for the three spinal regions.

**Results:**

The relative proportions of individuals with pain ever, who also reported to have had pain in the past year varied between 75% and 80%, for the three spinal regions. The proportions of individuals with pain in the past year and for various pain durations were also very similar. Regardless if pain was reported in the lumbar, thoracic or cervical regions, the proportions of individuals reporting radiating pain were equally large. The relative number of consequences was the same across the spinal regions, as were the relative proportions of each these consequences. However, low back pain resulted more often in some kind of consequence compared to the consequences of pain in the neck and mid back.

**Conclusions:**

Back pain and its consequences share many characteristics and may, at least in a general population, be regarded as the same condition regardless of where the pain happens to manifest itself. However, because some exceptions were noted for the lumbar spine, separate entities for a smaller group of individuals with back pain cannot be ruled out.

## Background

Traditionally, studies on non-specific back pain are focused on a single spinal region, such as low back pain (LBP), mid-back pain (MBP) or neck pain (NP). This approach may be based on the assumption that pain in different spinal regions are distinctive entities and that the prevalence and characteristics of pain in each of the three major regions vary considerably. The majority of studies are focused on LBP, as it seems to be the most prevalent spinal disorders, followed by NP, whereas far fewer studies are dealing with MBP. Recently, however, the idea that non-specific back pain is a site-specific disorder has been challenged, suggesting that localized musculoskeletal pain should be seen as part of a more general musculoskeletal syndrome [1,2].

Several arguments support this suggestion: First, there is a large degree of co-occurrence in musculoskeletal diseases, as a large proportion of people with musculoskeletal problems have pain in more than one site [1,3-7]. Second, many of the non-specific musculoskeletal pain syndromes share common factors with each other [2,5,8,9]. Third, the one-year transition pattern of reported pain has been noted to be fairly similar in different spinal regions [10]. Finally, the genetic contributions of pain in different spinal regions are fairly consistent, which suggests that there may be a common genetic basis for back pain in general. All these facts suggest that pain in different spinal regions should not be regarded as separate disorders but rather that back pain - regardless of location - may be a single entity.

In order to determine if back pain is a single entity or not it would be necessary to compare the different regions with each other using a large population-based cohort. However, such studies are lacking as most studies report only single spinal regions. The objective of this paper is therefore to report on the patterns of non-specific pain in each of the three spinal regions and their consequences in order to determine the degree of similarity or difference between the regions.

## Methods

### Study design and validity of data

The data were obtained from the 2002 Danish national twin survey. In this study, all twins born between 1931 and 1982 (i.e. aged 20 to 71), who had previously consented to take part in research (N = 46,818), were sent a 20-page health related questionnaire. The information letter stated that the project was focusing on twins' health in general. The questionnaire was followed by one reminder, which is the number of reminders allowed by the Danish Scientific Ethical Committees. The study had the required permissions from the Regional Scientific Ethics Committee and the Danish Data Protection Agency (file number: 20010201).

The twin cohort and the present study population are representative of the Danish population in terms of various diseases such as diabetes, nickel allergy and psoriasis [11]. In addition, the mortality rate is similar to that in the general population [12]. Also, the present study population was found to be similar to the Danish population for the most common sociodemographic variables and differences between responders and non-responders were similar to what is usually found in epidemiologic surveys (i.e. younger, single males not in a full-time employment situation were somewhat more likely not to respond) [13]. A sub-sample of this study population has previously been shown to have a one-year period prevalence of low back pain (LBP) corresponding to the best estimates of LBP in other Nordic epidemiologic population-based studies [14]. We were therefore confident that the present study sample is relatively representative of the general Danish population aged 20 to 71 years both in general and in relation to back pain.

### Data collection and variables of interest

A one-page questionnaire was included within the large survey, with identical questions asked, independently, for each of the three spinal regions at a time, in relation to pain and consequences. Questions on the three spinal regions were accompanied by drawings showing the anatomical boundaries of the lumbar, thoracic and cervical regions, respectively. Thus the participants were forced to reflect on pain and consequences for each spinal region separately. Questions were based on the Standardised Nordic Questionnaire [15].

The following variables were included: Pain ever, pain in the past year, number of days with pain in the past year (categorised as "≤ 30 days" and "> 30 days"), and pain radiating from the region of complaint (i.e. into the leg, chest, or arm). In relation to consequences of back pain during the past year, the following independent variables were used: 'care seeking', 'reduced physical activity', 'sick-leave', 'changed work/work duties', and 'seeking/being on disability pension'.

### Analysis and presentation of data

Data cleaning was carried out prior to the data analysis and resulted in less than 1% missing data for the individual pain and consequence-variables [16]. Descriptive data are presented for the whole study sample with emphasis on 1) *back and radiating pain for each region *and 2) *consequences of back pain for each of the three regions*. The relative frequencies of findings were calculated in relation to each of the different pain regions, including the relative proportions of individuals with radiating pain. For example, the proportion of individuals with pain radiating into the leg was calculated in relation to the number of individuals with pain in the lumbar region, and the proportions of individuals with pain radiating into the chest or arm were calculated in relation to the numbers of individuals with pain in the thoracic or neck regions, respectively. In addition, the relative proportions of consequences were calculated for each of the different regions of pain. The proportion of subjects described as 'changed work/work duties' or 'seeking/being on disability pension' were based on the 'pain ever' variable.

Previous analyses of prevalence of pain and its consequences showed remarkable similarities across the ages and only small non-significant differences between genders [16,17]. For this reason, and in order to obtain a sufficient number of individuals in each sub-category, data were reported for the whole study sample without taking into account the effect of age or gender. Because of the large study sample, 95% confidence intervals were generally very narrow (typically ± 1%) and thus not reported.

## Results

### Descriptive data

In all, 34,902 (74%) responded after the reminder with more women participating in the study (54.5%). A detailed description of the study sample can be found in our previous publications on the prevalence of back pain [16], the consequences on back pain [17], and on the genetic epidemiology on back pain [18].

## The relative frequencies of back pain

### Prevalence of back pain

As can be seen in Table 1 the prevalence estimates of LBP, MBP and NP differ considerably; with LBPever and LBPyear being the most frequently reported disorder followed by NPever and NPyear. MBPever and MBPyear were least common.

**Table 1:** Prevalence estimates of different definitions of back pain (N = 34,902)

	n	(%)
**LBP**ever	20,053	(57)
**LBP**year	15,093	(43) *
LBP < 8 days	3,804	(10)
LBP 8-30 days	6,168	(18)
LBP > 30 days	4,207	(12)
**Radiating pain into leg(s)**	7,651	(22)
**MBP**ever	5,966	(17)
**MBP**year	4,535	(13) *
MBP < 8 days	1,161	(3)
MBP 8-30 days	1,633	(5)
MBP > 30 days	1,338	(4)
**Pain radiating into chest**	1,846	(5)
**NP**ever	14,059	(40)
**NP**year	11,316	(30) *
NP < 8 days	2,523	(7)
NP 8-30 days	4,345	(12)
NP > 30 days	3,641	(10)
**Pain radiating into arm**(s)	5,583	(16)

*The numbers of positive replies in the day intervals do not add up to the year-estimates, because the latter have been corrected, if data were missing, based on subsequent answers in relation to site-specific consequences in the past year.

Pain in more than one region was very common and only 22% of the whole study sample had had back pain at a single region only in their life time.

### Back pain in the past year in relation to back pain ever

**Figure 1:**
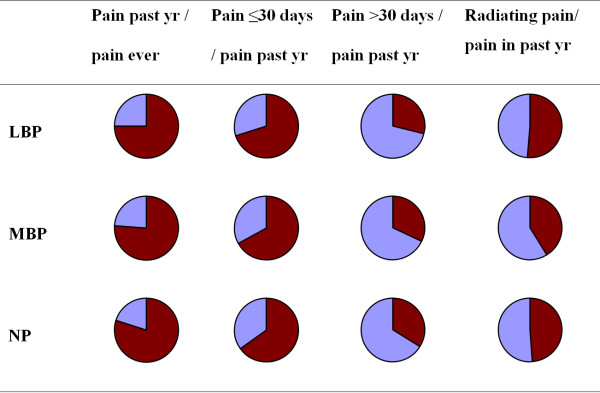
**Relative proportions of pain by region of back pain**. Pie diagrams of the relative proportions of people reporting pain by region of back pain in relation to pain in the past year or pain ever. The dark red areas represent the percentage of individuals with back pain or radiating pain.

The proportions of those having had back pain the past year among those who reported having had back pain ever were fairly similar for the three spinal regions ranging between 75% (for LBP) and 80% (for NP) (Figure 1, first column).

Also, those who recalled having had back pain in the past year had been similarly affected by radiating pain emanating from the region of complaint, regardless of the spinal region of complaint (Figure 1, last column)

### Number of days with back pain in relation to back pain in the past year

The relative frequencies for each of the back pain periods were similar in all three spinal regions regardless of pain duration (Figure 1, columns 2 and 3).

### Consequences of pain in relation to back pain in the past year

#### The proportions of number of consequences in relation to region of back pain

**Figure 2:**
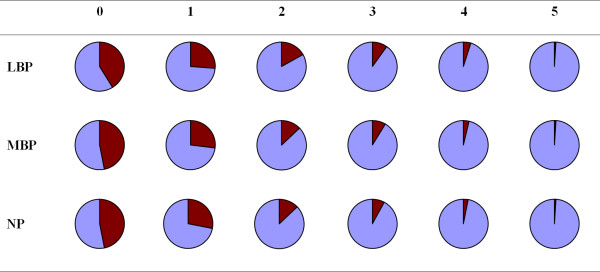
**Distribution of consequences of back pain**. Pie diagrams of the distribution of consequences by region of back pain within the past year. The dark red areas represent the percentage of individuals with consequences of back pain.

Overall, the proportions of number of consequences in relation to the reporting of back pain were similar regardless of the pain site (Figure 2). The pattern did not change with increasing pain duration (data not shown).

#### Consequences of back pain from these regions

**Figure 3:**
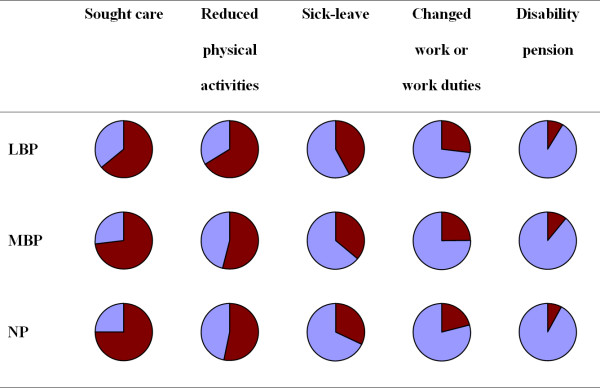
**Relative proportion of consequences of back pain**. Pie diagrams of the relative proportion of consequences of back pain in relation to each region of back pain within the past year. The dark red areas represent the percentage of individuals with consequences of back pain.

Figure 3 shows the relative proportions of each consequence for all three spinal regions. The relative proportions of all five consequences showed a similar hierarchy regardless the region of pain, with 'care seeking' being the most common choice followed by 'reduction in physical activities', 'sick-leave', 'change work', 'disability pension'. The relative proportions of consequences for NP and MBP were almost identical, whereas people with LBP had somewhat higher proportions of consequences, except for care-seeking, compared to the other spinal regions.

#### Consequences of back pain in relation to duration of pain in the past year

People with LBP were generally somewhat more likely than others to report consequences irrespectively of the pain duration, except for 'care-seeking' in which NP and MBP were somewhat more commonly reported. However, the same hierarchy and patterns of consequences were noted regardless of the pain duration (data not shown).

## Discussion

The results of this study demonstrate that although LBP is the most prevalent complaint in the general population, the relative proportions of people with back pain, including radiating pain, and the relative proportions of people reporting consequences thereof, are similar in all three spinal regions. It is particularly interesting that the relative percentage of people with pain in a spinal region, who also report to have had radiating pain from that particular region, is almost identical despite anatomical, functional and symptomatic differences in the three regions.

Our findings suggest that there are no obvious or unique pain patterns for individual spine regions, at least not on the variables that we studied. Rather, these similar pain patterns may reflect a general expression of pain and if this is correct then this distribution may also be found in other musculoskeletal pain syndromes. A literature search revealed two Nordic studies of the general population, in which pain data could be extrapolated in a similar manner. In the first study, based on 850 adults from Iceland [19], pain in the past year and in the past week were reported for the neck, upper back, and low back, and all major joints in the body. The proportions of people who had experienced pain in the past week out of those who reported to have had pain in the past year were remarkably similar (about 50%) for six of their nine musculoskeletal sites. In the second study, based on 46,901 Norwegian adults [20], the duration of pain in the past month was reported as < 15 days for about 1/3 of the study sample, regardless if pain was noted for the neck, upper back or low back. The same proportion was found also for the hips, knees, ankles/feet, elbows, and wrist/hands.

We also found similarities in relation to the consequences of back pain. For all three regions of the spine, slightly more than half of those who had experienced pain during the past year also reported some type of consequence of the pain. The majority reported one or two consequences, which were typically care-seeking or reduced physical activities. Regardless of the region and duration of the pain, the hierarchy of consequences was remarkably similar. Although, the hierarchy of the five consequences demonstrates a logical preference of choices for any musculoskeletal pain, it is nevertheless striking how the relative proportions are almost identical for the three spinal regions even with longer pain durations. This, too, indicates that the pattern of reactions may have some common mechanisms or expressions based on similarity in the condition or a similarity in how people react to spine-related pain regardless of where it hurts.

Despite the obvious similarities for all three spinal regions, some variations were noted. In particular, LBP resulted in relatively more consequences compared to MBP and NP. This is in accordance with other studies, in which especially sick-leave and care seeking are more commonly reported in people with LBP [19]. These findings indicate either that problems in the lumbar spine affect people's life more than problems in the neck/mid back or that there is a subgroup of people with LBP, who have a different type of condition, and that this type of LBP creates more problems than non-specific pain in the neck or the mid back.

### Strengths and limitations

This study was conducted on a large Danish cohort of twins that has been shown to be representative of its background population [13,21]. Although the twins received a 20-page long questionnaire, the response rate was rather high (74%), which gives this study a strong external validity. As the questionnaire entailed a large number of other health related issues, we have no reasons to suspect any 'distortions' of our data, as it would not have attracted specifically people with back problems. Finally, a previously validated questionnaire was used [15]. So all in all, our results are not likely to be biased in any major way.

It is relevant to note that there are no financial barriers to access the Danish health-care system and that sick-leave is available regardless of the cause of the disease. Hence, people's choice of consequences in this study was not based on financial issues to any significant extent. However, individuals in countries with other health care systems may of course be subjected to other constraints, which may affect the hierarchy of consequences.

Still, this was a cross-sectional study and it is therefore impossible to study causality and the order of events (e.g. in relation to the consequences). Additionally, any specific diagnoses cannot be determined in this cohort. However, people with LBP in the general population would be classified as having non-specific back pain. Therefore, the issue of diagnosis becomes irrelevant. Additional strengths and limitations of the study have been discussed in a previous publication based on the same study sample [16,17].

While our study showed many similarities between the spinal regions, one needs to keep in mind that there would be a fair degree of concurrent pain sites [1,3-7]. Thus, it is possible that memories of pain and its consequences may be confused between the different spinal regions. The next question is therefore if those with multi-site back pain differ from those with localized back pain in terms of the reported pain patterns and consequences. Hence, a follow-up on this study will be a comparison between widespread back pain and localized back pain. This way, it will be possible to determine if our main results can be reproduced.

## Conclusions

Remarkably similar patterns in pain reporting and its consequences of pain were noted for the three spinal regions. We therefore propose that, at least in a general population, back pain in terms of its relative prevalence and consequences may be regarded as the same condition regardless of where the pain happens to manifest itself. However, some exceptions were noted, particularly in relation to pain in the lumbar spine and further studies are needed to confirm our main results.

## Competing interests

The authors declare that they have no competing interests.

## Authors' contributions

All authors read and approved the final manuscript. KOK was responsible for the epidemiologic study. JH, RF and CLY secured funding for the back pain study. CLY and JH formulated the preliminary research questions and designed the back pain questionnaire. CLY formulated the research questions for the present analyses. JN and RF analyzed the data and RF provided the graphical presentations. CLY and RF did the data interpretation. CLY and RF wrote the first draft and all contributed to the final version.
